# The Investigation of the Structure and Properties of Ozone-Sterilized Nonwoven Biopolymer Materials for Medical Applications

**DOI:** 10.3390/polym13081268

**Published:** 2021-04-13

**Authors:** Polina Tyubaeva, Anna Zykova, Vyacheslav Podmasteriev, Anatoly Olkhov, Anatoly Popov, Alexey Iordanskii

**Affiliations:** 1 Department of Chemistry and Physics, Plekhanov Russian University of Economics, 117997 Moscow, Russia; zykovaak@yandex.ru (A.Z.); aolkhov72@yandex.ru (A.O.); anatoly.popov@mail.ru (A.P.); 2 Emanuel Institute of Biochemical Physics, Russian Academy of Sciences, 119991 Moscow, Russia; vpodmasterev@yandex.ru; 3 N.N. Semenov Federal Research Center for Chemical Physics, Russian Academy of Sciences, 119991 Moscow, Russia; iordan@chph.ras.ru

**Keywords:** ozone, polyhydroxybutyrate, electrospinning, supramolecular structure, nanocomposites, biomedical application

## Abstract

Nowadays, the development and research of nonwoven medical fibrous materials based on biopolymers is an area of a great practical interest. One of the most promising methods for producing nonwoven materials with a highly developed surface is electrospinning (ES). In this article, the possibility of efficient sterilization of ultrathin fibers based on polyhydroxybutyrate (PHB) by ozone treatment was considered. The purpose of this work was to select the most optimal morphology of nonwoven materials for medical purposes and to establish the correlation between the supramolecular structure and the physical properties of fibrous materials while under the influence of an ozone sterilization process.

## 1. Introduction

Nowadays, in biomedicine and food packaging industry, highly porous biodegradable polymers are widely used as long-acting functional matrices, scaffolds for tissue engineering, antibacterial barriers, and controlled release therapeutic systems, etc. [1,2,3,4,5,6,7,8]. Ultrathin fibrous materials with extremely high porosity can be obtained by various techniques, but within the framework of this paper, the greatest attention should be paid to the method of electrospinning (ES) via the formation of fibers from polymer solutions [9,10,11]. ES has already proven itself as an effective way to produce continuous fibers with a large variation in diameter [12,13]. The diversity of ES techniques for solution or melt modes enables modifying the fiber surface morphology and controlling the functional behavior of the resulting micro- and nanomaterials [14,15].

Nonwoven fibrous electrospun materials based on polyhydroxybutyrate (PHB) have a highly developed surface and a complex structure of ultrathin fibers. High porosity, large surface area, controlled biodegradation, and bioresorption in living organisms or environmental conditions are the advantages of these materials. However, the problem of the sterilization of these materials before usage in medical implants and food packaging is a topical issue in polymer material science. From a practical point of view, there is an essential interest in the study of ozone effect on materials and medical devices, because ozonolysis is one of the most effective techniques for sterilizing and purifying implants and packaging films [16,17,18].

In this work, the evolution of PHB structures under the influence of ozone was investigated, which allowed the authors to vary the properties of fibrous materials based on the biopolymer PHB to fabricate materials for biomedical applications. The controlled supramolecular structure of this materials allows controlling a number of key characteristics, for instance, the rate of biodegradation, modulus of elasticity, chemical stability, and others [19,20,21].

## 2. Materials and Methods

Nonwoven fibrous materials, which were obtained by the ES method on a single-capillary laboratory unit with the capillary diameter of 0.1 mm, were used for the investigation. Molding of the materials was carried out from the forming solutions of the PHB polymer. PHB (grade 16F) was produced by bacteriological synthesis by BIOMER (Krailling, Germany), with a molecular weight of 2.6 × 10^5^ Da. PHB content in the solution was 7 wt. The selection of the conditions was dependent on the properties of the molding solutions, such as conductivity, viscosity, and homogeneity. The conditions of the ES process were as follows: the voltage was 20 kV, the distance between the electrodes was 170 mm, and the gas pressure on the solution was 10 kg(f)/cm^2^.

In order to characterize the amorphous regions of the fibrous materials, the electronic paramagnetic resonance (later EPR) spectra were recorded on an EPR-V automated spectrometer. To avoid saturation effects, the value of the microwave power in the resonator cavity did not exceed 7 mW. The stable nitroxyl radical TEMPO was used as a probe. The radical was introduced into the fibers from the gas phase at a temperature of 60 °C. The content of the radical in the polymer was determined using Bruker WinEPR and SimFonia software (CCl_4_ with a known radical concentration, which did not exceed 10^–3^ mol/L, served as a reference). The values of the correlation time of the probe rotation were obtained from the obtained EPR spectra.

The study of the thermal properties of the samples was carried out by using a differential scanning calorimeter (DSC), Netzsch 214 Polyma (Selb, Germany), in an air atmosphere, with a heating rate of 10 K/min. The samples were heated from 20 °C to 200 °C and then cooled to 20 °C. At the second cycle of the study the melting temperature, melting enthalpy, and degree of crystallinity was determined. The average statistical error in measuring thermal effects was ±2.5%. The enthalpy of melting was calculated using the NETZSCH Proteus software program. The mass of all samples encapsulated into aluminum pans was kept at about 10 mg.

The morphology and the mutual arrangement of fibers in the structure of the material was investigated using an optical polarizing microscope Micromed Polar 3 ToupCam 5.1 MP (Saint-Peterburg, Russia) in reflected light at 200-fold magnification.

Mechanical tests were performed using a machine for mechanical analysis DEVOTRANS (Istanbul, Turkey) in compliance with the ASTM D5035-11.

The chemical sterilization of nonwoven materials was conducted by the ozonation method, using a flow-through reactor. The ozone was synthetized from oxygen in a barrier discharge. The required ozone contents were set by changing the voltage at the ozonizing apparatus electrodes (5–7 kV). The ozone content was controlled spectrophotometrically at the wave length of 254 nm. The basic working ozone content was 5.5 × 10^−5^ mol/L. The duration of the ozonation was varied from 1 to 600 min. The rate of ozone expenditure was 0.1 L/min. The quantity of absorbed ozone was calculated as the difference between input and output ozone contents (in the presence of the specimen in the reactor), respectively.

## 3. Results

### 3.1. Morphology of the Fibrous Nonwoven PHB

**Figure 1:**
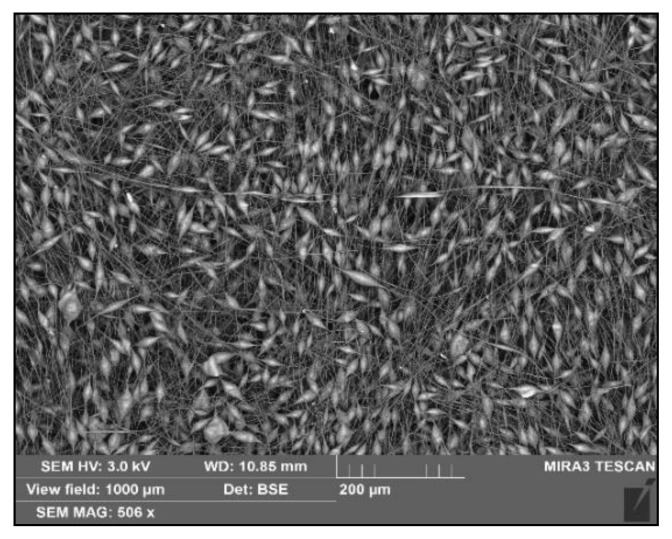
Microphotographs of the nonwoven polyhydroxybutyrate (PHB) obtained by the solution-mode electrospinning (ES).

The supramolecular structure of biopolymer electrospun fibers is highly sensitive, not only to the molecular characteristics of the polymer, but also to the operation conditions of fiber fabrication [19]. The porosity, geometry, and surface topology of a single filament, as well as their mutual orientation, make a significant contribution to the formation of the physical, mechanical, diffusion, and thermal properties of the whole material. It was established that the supramolecular structure of fibrous PHB predominantly determined the biodegradation rate of the material and its functional behavior, which could be improved and controlled by several effective ways, for instance, by incorporating specific additives, chemical modification of fibers, and variation of technological conditions of the electrospinning (ES) at various stages of fiber fabrication [20,21]. Figure 1 shows the structure of the material, obtained by the earlier described ES method.

The specific structure of the fibers can be observed. The average diameter of the fibers varied in the range of 4–6 µm. Specific thickenings may be noticed on the surface of the fibers, with an average length 25–35 µm and with the average size at the widest part of 12–18 µm. The emergence of such thickenings can be explained by the low electrical conductivity of PHB solutions in the capillary.

Such kinds of nonwoven materials based on PHB are widely used in various medical applications, such as air filtration [22], drug delivery systems [23], biosensors [24], and bone, blood vessel, and skin tissue engineering [25], etc.

### 3.2. Sterilization

**Figure 2:**
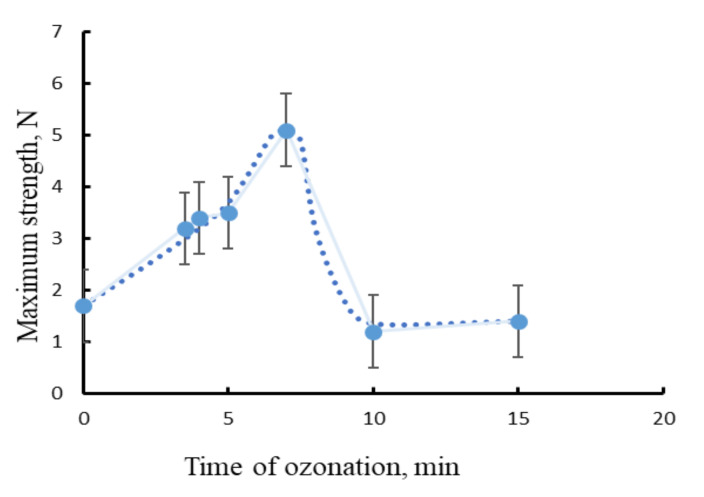
The correlation between PHB maximum strength and ozonation time.

As we mentioned above, the sterilization of fibrous materials before use in packaging and medical devices is the acute issue. Their highly developed surface makes it difficult to sterilize nonwoven materials by UV treatment. In addition, various studies have shown an uncontrolled decrease in the physical and mechanical properties of the materials based on the PHB after irradiation at a wavelength of 254 nm [26]. It is known that PHB is characterized by the initial temperature of destruction, which is 140–150 °C; 40–30 °C lower than the recommended temperature required for the death of pathogenic microorganisms. This fact makes high-temperature annealing inapplicable for nonwoven materials. The problem of sterilization can be solved by an effective method of disinfection by the ozone treatment [17,27]. The effect of ozone on the supramolecular structure of PHB fibers obtained by the described formulation was investigated in this section. Figure 2 presents the changes in the ultimate strength as a result of ozonolysis duration. It is seen that, during the initial stage of ozonation, a significant increase of ultimate tensile strength was detected. The highest value was reached by the 7th minute of ozone treatment.

It is worth noting that the growth of physical and mechanical parameters was recorded at the first minutes of ozonolysis and reached a maximum in the range of 3–5 min. Thus, it might be suggested that even at the early stage of ozonation, ozone could provide both effective sterilization of the material and a significant improvement of the physical and mechanical parameters. Table 1 shows the comparison of mechanical characteristics of PHB for the initial, and treated with ozonolysis, fibrous samples. Both samples were obtained by electrospinning at the same conditions. For the ozone-treated fibrous material, an increase in tensile stress and elongation at break compared to the analogous mechanical characteristics of the initial PHB was clearly observed. It is important that during this short period of ozone action the gaseous ozone penetrated into the bulk of the material and had the appropriate sterilizing effect.

**Figure 3:**
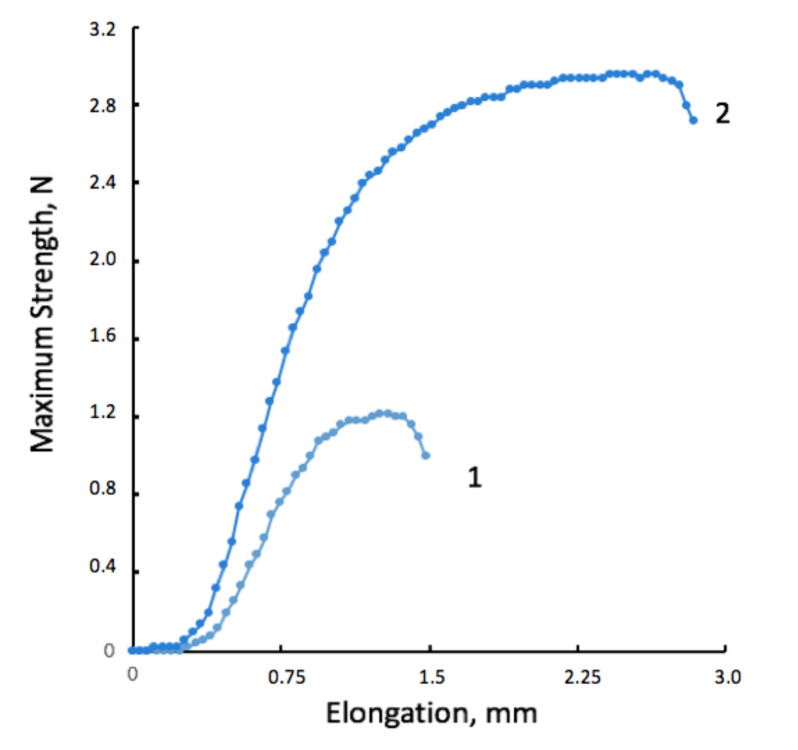
Stress–strain behavior diagram of PHB fibrous material before the ozonation (1), and after 5 min of ozonation (2).

A typical stress–strain diagram for the sample of the material based on PHB before the ozonation (1) and after 5 min of ozonation (2) is presented in Figure 3.

**Figure 4:**
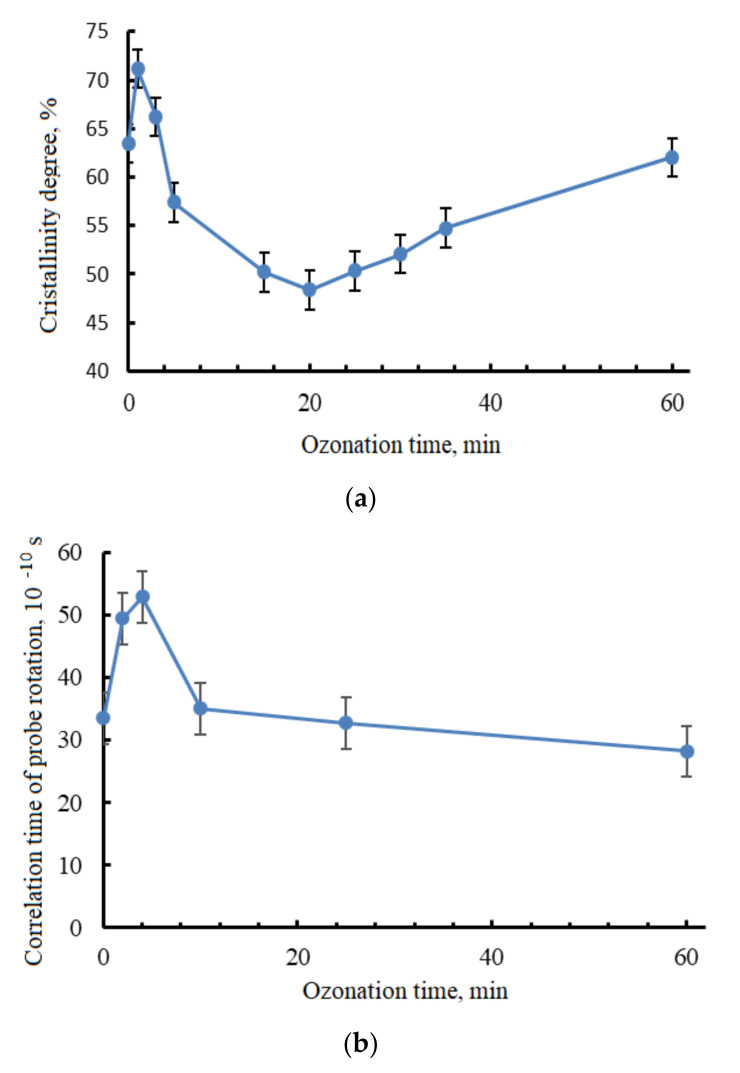
Crystallinity degree (**а**) and correlation time of probe rotation (**b**) in PHB, dependent on ozonation duration.

It is worth noting that the mechanical characteristics correlated to the degree of crystallinity and features of amorphous phase of PHB were determined by the EPR method, which can be seen in Figure 4a,b. It was found that the highest level of PHB crystallinity degree (Figure 4a) and correlation time of probe rotation, dependent on ozonolysis duration (Figure 4b), located approximately in the same time interval as the mechanical characteristic maximums presented in Figure 2 and Table 1.

### 3.3. Ozonolysis

Like oxygen, gaseous ozone has proved to be an extremely strong oxidizing agent, capable of significant influence on macromolecular structure, polymer morphology, and crystallinity [25,26]. Despite the significant advantages of ozone sterilization in comparison with oxygen, such as short a period of contact with the packaging materials and implants, the ozone impact on the structural and dynamic characteristics of different polymers has remained a poorly studied area of polymer science. According to the PLA ozonolysis model developed by Olevnik-Kruszkovska, the oxidizing agent affected the structure of the polymers in a complex way. First, the network formation occurred at favorable conditions, namely, elongated intercrystalline chains contacted each other in an amorphous polymer area of the fibrils. As long as ozonation continued, the destruction of the main polymer chain, accompanied by the decrease in averaged molecular weight, was observed, which resulted in an increase of segmental mobility due to removing conformational constraints. The increase in the crystallinity degree at the first stage of ozonolysis correlated with the idea of reinforcement of intercrystalline polymer molecules in the PHB amorphous phase. As the result of pendant groups’ oxidation and their following interactions, their orientation was enhanced and the crystallinity increased [28]. In the PHB fibers, the content of straightened macromolecules was the most sensitive characteristic to the ozone oxidation. Moreover, from earlier papers [29,30,31,32], it is known that “chemical relaxation” emerges during oxidation. This process involves relaxation of the most strained macromolecules located in the amorphous polymer phase. The mechanism of relaxation is similar to the thermal oxidation of polyolefins, but proceeds at a higher rate [33,34,35]. In the first minutes of ozonolysis, the most strained intercrystalline chains of PHB resulted in an increase of crystallinity degree, and following this, chain destruction could occur.

**Figure 5:**
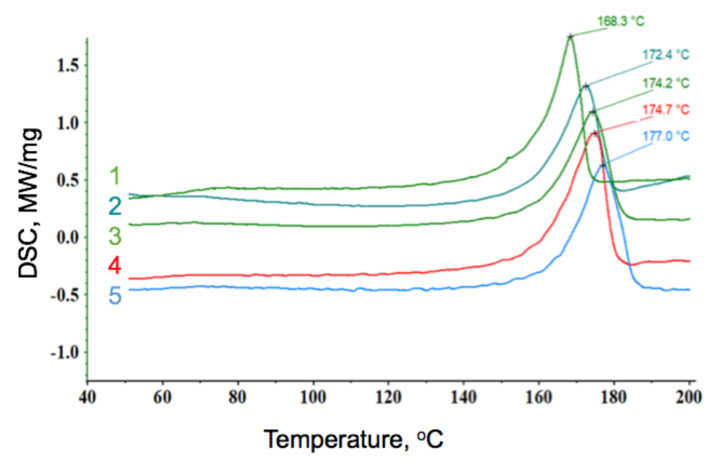
The differential scanning calorimeter (DSC) curves of the PHB, dependent on period of ozonation: 1—5 min of ozonation, 2—30 min, 3—60 min, 4—0 min, and 5—90 min.

Figure 5 represents the DSC curves of the materials. It shows the dependence of thermal parameters on the ozonation time. The PHB degree of crystallinity was determined as the ratio between the measured heat fusion of the sample, and the heat of fusion of a 100% crystalline PHB [33]. According to the data, it was concluded that a small ozonation period (curve 1) caused the most significant decrease of melting temperature and degree of crystallinity. The following ozone treatment led to irreversible polymer changes and degradation. This process began in the most reactive segments of the polymer; the chain folds and passing chains in particular. Such oxidized macromolecules began to break, which resulted in increased amorphous phase. It should be noted that amorphous regions of the polymer were highly reactive, as well as the chain folds and passing chains.

During the ozonation treatment, the chain-scission of polymer macrochains was observed. At the first stage, regular and long chains broke, as well as short ones. Later, the degradation of regular chains led to the shift of melting temperature to higher values. The character of the melting peak changed; in other words, it became broader.

## 4. Discussion

The increase of the degree of crystallinity during the first minutes of ozonation correlated with the condensation of the PHB amorphous phase, which occurred in the process of ES on the electrode. These two parameters, crystallinity and the density of the amorphous phase, were the most sensitive to the processes that occurred in the PHB supramolecular structure under the influence of such an aggressive oxidizing agent as ozone. Commonly, “chemical relaxation” in polymers is attributed to the oxidation of a polymer chain. This process involves relaxation of the most strained macromolecules, which are contained in the polymer structure, similar to the process of thermal oxidation of polyolefins [22,23], but occurring at a higher rate. During the first minutes of ozonolysis, the most strained passing chains of PHB, which completed the crystalline phase, contributed to the condensation and weakening of the amorphous areas. This process completely stopped after 7–10 min of ozonation, and a gradual degradation was observed.

**Figure 6:**
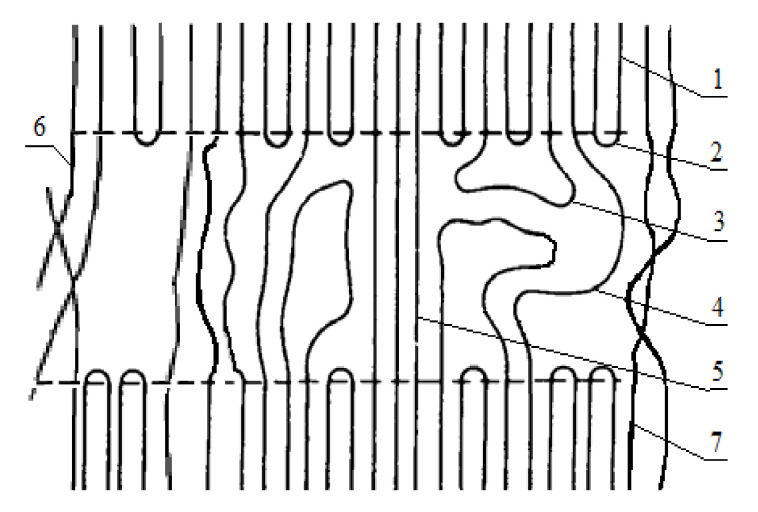
The structural organization of the PHB macromolecules, where: 1 = crystalline region; 2 = regular chain fold; 3 = irregular chain fold (free loop); 4 = passing macromolecules; 5 = the shortest, most elongated и strained passing macromolecules; 6 = interfibrillar chains macromolecules; and 7 = interfibrillar structures.

In this work, reasons for the observed effects were suggested, and it resulted in the macromolecules’ fracture in the amorphous phase followed by a more regular laying of macrochains of PHB. It is important to note that, at the moment of fiber formation, polymer macromolecules were subjected to orientation and extraction under the action of a complex of physical forces. Figure 5 shows the schematic image of PHB macromolecules after the ES process. The structure of PHB macromolecules can be described as oriented [31]. It is worth noting that there were a number of structural strains and non-equilibriums of crystalline and amorphous phases [32]. The highest strain was fixed in such areas as the chain folds at the border of crystallites (Figure 6, number 1) and these areas of passing chains (Figure 6, number 5).

It was observed that ozone reacted with the amorphous areas of PHB, then with the most strained and reactive chain sections.

The EPR method allowed seeing the effect of ozone on the amorphous phase of the polymer, where the reaction was quite intense. In the first minutes of ozonation, the correlation time of the probe increased from 30 × 10^−10^ to 60 × 10^−10^ s, which indicated changes in the amorphous phase of the PHB and its densification. The DSC method showed an accompanying increase in the degree of crystallinity, as well as an increase in the PHB melting temperature at 3–5 min of ozonation. This could be explained by both the scission of the most stressed sections of the polymer chain, and the reorganization of the amorphous phase sections, which contributed to a more ordered arrangement of macromolecules in the crystalline regions. Possibly, this phenomena, led to an increase in physical and mechanical properties in the first minutes of ozonation. Moreover, as soon as the ozonation time exceeded 7–8 min, the degradation of the material became visible, which is shown in Figure 4b as a drastic decrease of correlation time.

## 5. Conclusions

Various types of materials could be obtained under different conditions of solution-mode electrospinning. The characterization of ultrathin fibers and nonwoven materials after their effective sterilization is an area of a great interest. This article established and showed an example of an effective sterilization, which can improve the physical and mechanical characteristics of the material, and also considered the reasons for this process; due to the influence of ozone on the supramolecular structure of the material. According to the results of the series of conducted studies, it was concluded that the tensile strength of PHB nonwoven fibrous materials rose significantly under the influence of ozone. Moreover, the elasticity modulus, relative deformation, and maximum elongation of the material rose markedly before the moment of rupture. In this work, the possible reasons for such changes in the strength properties of the materials were studied. It worth noting that at the initial stage of ozonation, macromolecules broke up, and the possibility of more regular packing of molecules in the amorphous phase emerged. This was confirmed by a significant growth of melting enthalpy of the ozonized PHB samples. The obtained data emphasized the great potential of the usage of non-woven materials based on electrospun fibrous PHB for medical purposes, and confirmed the effectiveness of ozone sterilization of these materials, without the loss of mechanical properties.

## Figures and Tables

**Table 1:** Physical-mechanical properties of nonwoven materials before and after ozone treatment (duration of the ozone treatment was 5 min long).

Material	Maximum Strength, N (Δ ± 0.5)	Relative Deformation, % (Δ ± 0.2)
PHB Initial	1.7	3.4
PHB Ozonized	3.5	7.6

## Data Availability

The data presented in this study are available on request from the corresponding author.
